# Research Progress of Annexin A1 and Its Derived Peptides in the Diagnosis and Treatment of Circulatory Diseases

**DOI:** 10.1002/iid3.70249

**Published:** 2025-11-10

**Authors:** Qiuyu Dai, Jie Zheng, Meng Fu, Song Qin, Xiaoyun Fu

**Affiliations:** ^1^ The First Clinical College Zunyi Medical University Zunyi City China; ^2^ Department of Critical Care Medicine Affiliated Hospital of Zunyi Medical University Zunyi City China

**Keywords:** Annexin A1, diseases of the circulatory system, immunosuppression, inflammation

## Abstract

**Background:**

Annexin A1 (ANXA1), a calcium‐dependent phospholipid‐binding protein, plays a critical role in regulating inflammation, apoptosis, immune responses, and vascular remodeling. It is increasingly recognized for its potential as a therapeutic target and diagnostic biomarker in various circulatory diseases, including sepsis, hypertension, coronary heart disease, myocardial ischemia‐reperfusion injury, heart failure, and cerebrovascular disorders. ANXA1 and its derived peptides exert protective effects by modulating key signaling pathways and cellular processes involved in disease pathogenesis.

**Aim:**

This review summarizes the current research progress on ANXA1 and its derived peptides in the diagnosis and treatment of circulatory diseases, highlighting their mechanisms of action and therapeutic potential.

**Method:**

A comprehensive literature search was conducted using PubMed, Web of Science, and other relevant databases. Articles published up to 2024 were included, with a focus on experimental studies, clinical reports, and reviews addressing the role of ANXA1 in circulatory diseases. Key themes included anti‐inflammatory mechanisms, apoptosis regulation, immune modulation, and vascular protection.

**Result:**

ANXA1 and its mimetic peptides like Ac2‐26, ANXA1sp, RTP‐026 and so on demonstrate significant protective effects across multiple circulatory diseases. They attenuate inflammation, oxidative stress, apoptosis, and ferroptosis, while promoting mitochondrial biosynthesis and immune regulation. These effects are mediated through receptors such as FPR2/ALX and pathways involving SIRT3, PI3K/Akt, and AMPK/mTOR. However, the role of ANXA1 can be context‐dependent, exhibiting both protective and detrimental effects in specific conditions such as gestational hypertension and transient ischemic attack.

**Conclusion:**

ANXA1 represents a promising diagnostic biomarker and therapeutic target for circulatory diseases. Further research is needed to elucidate its complex regulatory networks and validate its clinical applicability through multi‐omics approaches and large‐scale trials.

## Summary

- ANXA1 regulates inflammation, apoptosis, and immune responses in circulatory diseases.
- Derived peptides mimic ANXA1's bioactivity and show therapeutic potential.
- ANXA1's role is context‐dependent, requiring disease‐specific investigation.
- Future studies should integrate omics technologies and clinical validation to optimize ANXA1‐based interventions.

**Abbreviations**
Arg‐1: arginase 1
ATP: adenosine 5′‐triphosphate
Bax: BCL2‐Associated X
Bcl‐2: B‐cell lymphoma‐2
BDNF: brain‐derived neurotrophic factor
Bid: Bcl‐2 homology 3 interacting domain death agonist
Ccl2: C‐C motif chemokine ligand 2
CD11b: CD11 antigen‐like family member B
CD16/32: Fc gamma III/II receptor
CD36: cluster of differentiation 36
CK‐MB: creatine kinase muscle/brain subtype
CMPs: common myeloid progenitors
COX2: cyclooxygenase‐2
cPLA‐2: cytosolic phospholipase A2
CSE: cystathionine gamma‐lyase
CTGF: connective tissue growth factor
cTnI: cardiac troponin I
CX3CL1: C–X3–C motif chemokine ligand 1
CX3CR1: C–X3–C motif chemokine receptor 1
Cxcl1: C–X–C motif chemokine ligand 1
DHE: dihydroethidium
DUSP1: dual specificity phosphatase‐1
eNOS: endothelial nitric oxide synthase
FADD: Fas‐associated with death domain protein
FPR2/ALX: formyl peptide receptor 2
FTH1: ferritin heavy chain 1
GATA‐3: GATA binding protein 3
Gpx: glutathione peroxidase
GSH: glutathione
HSP70: heat shock protein 70
HSPC: hematopoietic stem/progenitor cell
Iba‐1: Ionized calcium binding adaptor molecule 1
IFNγ: interferon‐γ
IKKα: inhibitory Kappa B Kinase α
IL‐10: interleukin‐10
IL‐1β: interleukin‐1β
IL‐4: interleukin‐4
IL‐6: interleukin‐6
iNOS: inducible nitric oxide synthase
JNK: c‐Jun N‐terminal kinase
JunB: Jun B proto‐oncogene
KC: keratinocyte‐derived chemokine
LC3: microtubule‐associated protein 1 light chain 3
LDH: lactate dehydrogenase
LXA4: lipoxin A4
MAPK: AMP‐activated protein kinase
MDA: malondialdehyde
MEP: megakaryocyte–erythroid progenitor cell
MIP‐1α: macrophage inflammatory protein‐1 alpha
MKP‐1: mitogen‐activated protein kinase 1
MMPs: matrix metalloproteinases
MMP‐9: matrix metalloproteinases 9
MPO: myeloperoxidase
MYH11: myosin heavy chain 11
MYL9: myosin regulatory light chain
NLRP3: the nucleotide‐binding domain and leucine‐rich repeat protein 3
PARP: poly ADP‐ribose polymerase
PGC‐1α: peroxisome proliferator‐activated receptor‐gamma coactivator‐1alpha
PGE2: prostaglandin E2
PGF2α: prostaglandin F2α
PKC: protein kinase C
PMN: polymorphonuclear neutrophil
PNA: peptide nucleic acids
PPAR‐γ: peroxisome proliferator‐activated receptor γ
PSD95: postsynaptic density protein‐95
p‐Akt/PKB: phosphorylated protein kinase B
p‐AMPAα: Phospho‐AMPKα
p‐AMPK: phosphorylated AMPK
p‐ERK: phosphorylated extracellular regulated protein kinases
p‐JAK2: phosphorylated Janus Kinase 2
p‐MTOR: phosphorylated mammalian target of rapamycin
p‐PI3K: phosphorylated phosphoinositide 3‐kinase
p‐STAT3: phosphorylated signal transducer and activator of transcription 3
Rap1: ras‐related protein 1
ROS: reactive oxygen species
SIRT3: sirtuin 3
SIRT3: sirtuin 3
SM22α: smooth muscle protein 22‐α
SMA: smooth muscles actin
SOD: superoxide dismutase
TFAM: mitochondrial transcription factor A
TNF‐α: tumor necrosis factor‐α
TpxⅡ: targeting protein for Xklp2
TRPM7: transient receptor potential cation channel subfamily M member 7
TXA2: thromboxane A2
T‐bet: T‐box expressed in T cells
VEGF: vascular endothelial growth factor
YM1: chitinase‐like protein 3
αIIbβ3: glycoprotein IIb/IIIa

## 1 Introduction

Annexin A1 (ANXA1) is a 37‐kD, 346‐amino acid intracellular protein encoded by the ANXA1 gene located on human chromosome 9 and is found in the nucleus, cytoplasm, and plasma membrane of various cell types [1]. Structurally, ANXA1 consists of a C‐terminal core region shared by the Annexin superfamily and an N‐terminal domain with unique functions; its structure plays a key role in determining its functional properties. ANXA1 contains several calcium‐binding sites in its C‐terminal core region. ANXA1 interacts with phospholipids in a calcium‐dependent manner and plays a crucial role in biofilm formation, ion channel establishment, signal transduction, and anti‐inflammation. Meanwhile, its N‐terminal domain contains regulatory regions of phosphorylation and proteolytic sites, which serve as targets for various signal‐transduction kinases [2].

ANXA1 has been confirmed to be involved in various physiological processes. Previous studies have reported that ANXA1 can down‐regulate the expression levels of cleaved caspase‐3, cleaved caspase‐8, FADD, and BAX to inhibit apoptosis [3]. Moreover, ANXA1 also regulates the function of platelets by downgrading integrin (αIIbβ3) to reduce platelet aggregation and thrombosis [4]. In addition, ANXA1 participates in oxidative stress and immune inflammatory response. A number of studies have confirmed that ANXA1 regulates neutrophil function and reduces their aggregation [5]; furthermore, ANXA1 promotes the transformation of macrophages to an anti‐inflammatory phenotype, affecting the expression of IL‐10, IL‐6, tumor necrosis factor‐α (TNF‐α), IL‐1β, and other related inflammatory factors, thereby inhibiting inflammation [6]. The effects of ANXA1 intervention on oxidative stress were evidenced by the changes in superoxide dismutase (SOD), malondialdehyde (MDA), and reactive oxygen species (ROS) levels [7]. Moreover, the immune effect of ANXA1 is closely related to the activation of CD4^+^ T cells [8]. Besides, ANXA1 has been found to inhibit vascular aging caused by chronic inflammation due to its anti‐inflammatory biological effects [9]. Xiao et al. [10] revealed that ANXA1 promoted tumor immune escape in cancer tissues by inhibiting the signal transducer and activator of transcription 3 (Stat3) protein pathway. Their findings suggest that ANXA1 competitively inhibits PARP1 binding to Stat3 by interacting with PARP1, which is an upstream regulator of the Stat3 protein. This interaction affects the ribosylation and dephosphorylation of poly (adenosine diphosphate‐ribosylation) on Stat3 protein and ultimately enhances Stat3 transcriptional activity. These changes lead to the upregulation of PD‐L1 transcriptional expression in multiple cancer cells and promote tumor immune escape).

To further explore the function of ANXA1, researchers created derived peptides and mimic peptides that have similar biological effects to ANXA1. These peptides include Annexin tripeptide (ANXA1sp), RTP‐026, CR‐Ac_2‐48_, CR‐Ac_2‐50_ [11], and Ac2‐26 [12], which is a mimic peptide formed by unfolding the full‐length ANXA1 protein and cutting off the N‐terminal 26 amino acids. At present, many ANXA1‐related experiments have been conducted using derived peptides [13, 14], confirming their similar biological effects [15] (Table 1).

**Table 1:** Sequence and main functions of ANXA1 and its main derived peptides.

The protein	Sequence	Main functions
ANXA1	MAMVSEFLKQAWFIENEEQEYVQTVKSSKGGPGSAVSPYPTFNPSSDVAALHKAIMVKGVDEATIIDILTKRNNAQRQQIKAAYLQETGKPLDETLKKAL	1. Plays important roles in glucocorticoid‐mediated downregulation of the early phase of the inflammatory response as effector of glucocorticoid‐mediated responses and regulator of the inflammatory process [16].
	TGHLEEVVLALLKTPAQFDADELRAAMKGLGTDEDTLIEILASRTNKEIRDINRVYREELKRDLAKDITSDTSGDFRNALLSLAKGDRSEDFGVNEDLAD	2. Contributes to the adaptive immune response by enhancing signaling cascades that are triggered by T‐cell activation, regulates differentiation and proliferation of activated T‐cells [17].
	SDARALYEAGERRKGTDVNVFNTILTTRSYPQLRRVFQKYTKYSKHDMNKVLDLELKGDIEKCLTAIVKCATSKPAFFAEKLHQAMKGVGTRHKALIRIM	3. Negatively regulates hormone exocytosis via activation of the formyl peptide receptors and reorganization of the actin cytoskeleton [18].
	VSRSEIDMNDIKAFYQKMYGISLCQAILDETKGDYEKILVALCGGN	4. Has high affinity for Ca^2+^ and Plays a role in phagocytosis by mediating the Ca2^+^‐dependent interaction between phagosomes and the actin cytoskeleton [19].
Ac2‐26	Ac‐AMVSEFLKQAWFIENEEQEYVQTVK	1. Functions at least in part by activating the formyl peptide receptors and downstream signaling cascades [20].
		2. Promotes chemotaxis of granulocytes and monocytes via activation of the formyl peptide receptors [21].
		3. Promotes rearrangement of the actin cytoskeleton, cell polarization and cell migration [21].
		4. Promotes resolution of inflammation and wound healing [20].
		5. Acts via neutrophil N‐formyl peptide receptors to enhance the release of CXCL2 [22].
ANXA1sp	Ac‐GAT	1. upregulates SIRT3, leading to the deacetylation of p53, thereby inhibiting cellular ferroptosis [23].
		2. upregulates SIRT3 and activates mitochondrial function [24, 25].
		3. inhibits oxidative stress and alleviates inflammation [24, 26].
		4. inhibits cell apoptosis and autophagy [24, 27].
RTP‐026	AMVSGPLLGATPIGAGGGGTVGTLLSSLGGPGSAVSPTPTPAPSSAV‐NH2 acetate salt	1. promotes acute cardio protection through modulation of immune cell activation [28].

**Figure 1:**
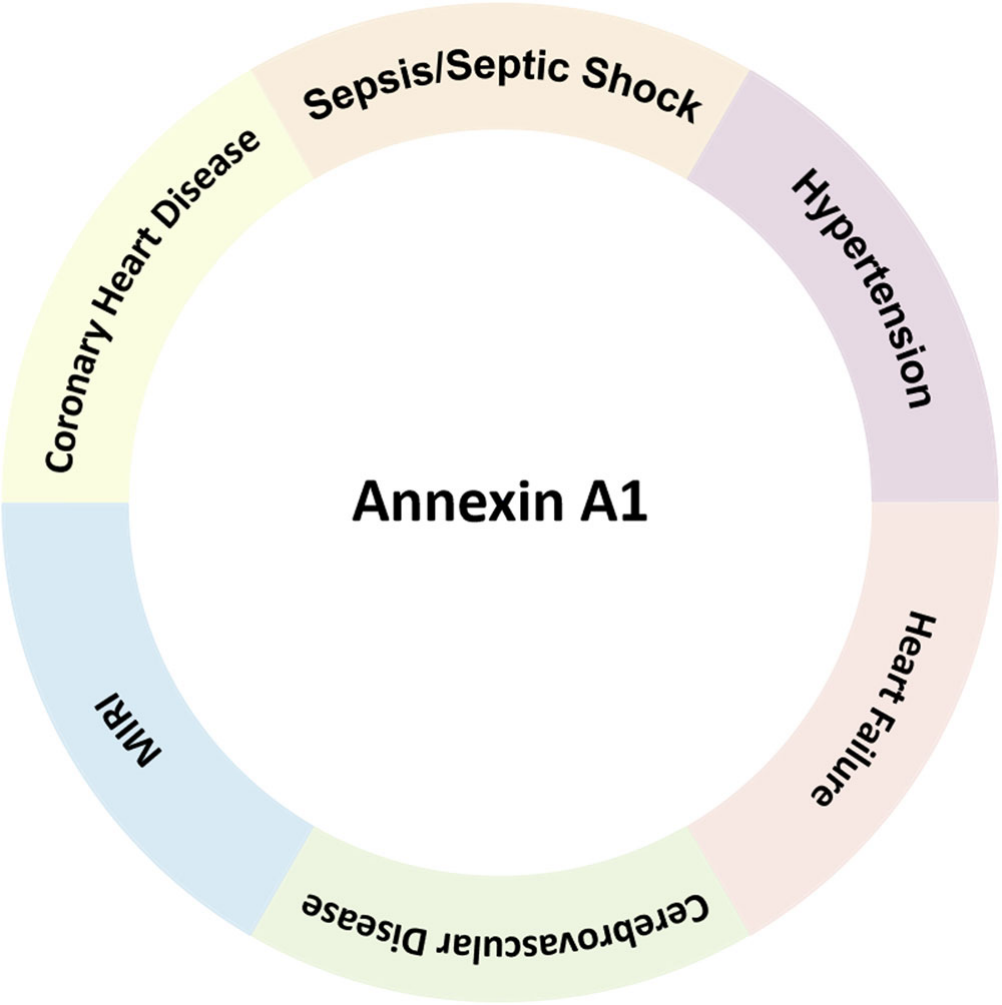
Annexin A1 and diseases of the circulatory system. The sequence of ANXA1: MAMVSEFLKQAWFIENEEQEYVQTVKSSKGGPGSAVSPYPTFNPSSDVAALHKAIMVKGVDEATIIDILTK. RNNAQRQQIKAAYLQETGKPLDETLKKALTGHLEEVVLALLKTPAQFDADELRAAMKGLGTDEDTLIEILASRTNKEIRDINRVYREELKRDLAKDITSDTSGDFRNALLSLAKGDRSEDFGVNEDLAD.

The purpose of this review is to summarize the research status of ANXA1 in circulatory diseases, to provide some theoretical support for the development of related experiments in the future (Figure 1).

## 2 ANXA1 and Sepsis/Septic Shock

Sepsis and septic shock are leading causes of death in critically ill patients [29]. Sepsis refers to a life‐threatening organ dysfunction caused by a dysregulated host immune response to infection [30]. Organ failure, including the heart, brain, and lung, is mainly caused by the host's excessive response to infection [31], is a serious complication of sepsis [29] and has high mortality. With the improvement of medical technology, recent research has proposed relevant pathological mechanisms. Many substances and mechanisms appear to be involved in organ dysfunction in patients with sepsis, including apoptosis, abnormal release of inflammatory mediators, mitochondrial dysfunction, oxidative stress, abnormal calcium regulation, and disorders of energy metabolism [32].

There are various indications that ANXA1 is closely related to the occurrence and development of sepsis. In 2005, a study found that ANXA1 in mouse epithelial cells, macrophages, as well as polymorphonuclear neutrophil (PMN) and monocytes could be activated by lipopolysaccharide (LPS), thereby inhibiting the elevation of organ damage markers in mice, producing a certain protective effect and reducing mortality. But the specific mechanism was still unclear at that time [33]. In 2014, When the researchers examined the process of changes in the plasma levels of ANXA1 and cortisol in patients with sepsis, they found that there was a subgroup of sepsis patients who were not affected by peripheral blood cortisol levels that had significantly increased plasma levels of ANXA1 [34], suggesting that ANXA1 may be a potential target for diagnosis and treatment of sepsis. In 2021, Chen et al. [35] also found that the ANXA1 gene was upregulated in sepsis patients compared to normal people during the period of constructing drug‐gene‐therapy‐cell network.

Therefore, a series of experimental studies was conducted to clarify the potential role of ANXA1, a potent anti‐inflammatory molecule, in the diagnosis and treatment of sepsis and septic shock. Huang et al. [8] reported that ANXA1 can induce the differentiation of CD4 T cells into Th0/Th1 and improve sepsis via immunosuppression by regulating GATA‐3 and T‐bet molecules through the extracellular regulated protein kinases (ERK)/protein kinase B (PKB/Akt) pathway activation. In 2024, Zhu et al. [36] studied the effect of ANXA1 gene knockout on sepsis‐associated encephalopathy model mice using single nuclear RNA sequencing and spatial transcriptomics. Compared to ordinary mice, the ANXA1 gene in gene knockout mice cannot be activated to produce ANXA1 protein. Their results showed that ANXA1 gene knockout mice exhibited higher mortality compared with wild‐type mice. After LPS injection, the proportion and distribution of Micro‐2 and Astro‐2 cells in the brain tissue of wild‐type mice were upregulated, forming a special V1A2M2 region surrounding Vas‐1 cells. The corresponding ligand and receptor pairs in this region were differentially expressed, which was significantly inhibited by the knockdown of ANXA1, resulting in higher mortality in mice.

Furthermore, derived peptides with similar biological effects to ANXA1 have also been studied in sepsis. Vital et al. found that the administration of Ac2‐26 could improve the septic patients prognosis by reducing the inflammation and inhibiting the formation of thrombosis [37]. In addition, Zheng et al. [3] conducted in vivo and in vitro experiments to confirm that Ac2‐26 binds to the receptor formyl peptide receptor 2 (FPR2) to inhibit inflammatory response and apoptosis to reduce sepsis‐induced acute kidney injury. In 2014, Gobbetti et al [38] discovered that treatment with the drug CR‐Ac_2‐50_ in wild‐type mice significantly improved cardiac damage induced by peritoneal sepsis and reduced inflammation levels (by inhibiting the recruitment of neutrophils and monocytes and downregulating the pro‐inflammatory factors TNF‐α and IL‐6); however, this effect was absent in FPR2/ALX gene knockout mice. This result indicates that the derived peptide CR‐Ac_2‐50_ from ANXA1 can inhibit the inflammatory response and improve sepsis, and this effect is achieved through the activation of the FPR2/ALX pathway. Qin also confirmed that ANXA1sp upregulated sirtuin‐3 (SIRT3), peroxisome proliferator‐activated receptor‐γ coactivator‐1α (PGC‐1α), and mitochondrial transcription factor A (TFAM). Its expression was found to improve mitochondrial dysfunction, inhibit oxidative stress, inflammatory response, and autophagy in septic myocardial injury [24]. On the other hand, upregulated SIRT3 molecule can also inhibit LPS‐induced ferroptosis of myocardial cells by acetylating the p53 molecule [23], which provides a new idea for the diagnosis and treatment of sepsis‐induced myocardial injury (Table 2).

**Table 2:** Possible mechanisms of ANXA1 and its derived peptides involved in septic/septic shock.

Administration	In vivo/in vitro model	Findings	Results	Reference
ANXA1	In vitro	HSP70, MKP‐1↑ MAPK, JNK, p38, TNF‐α ↓	Protective effect Inhibits autophagy and oxidative stress	Nair et al. [39]
	In vivo and in vitro	IFNγ, T‐bet, p‐ERK, p‐Akt↑ IL‐4, GATA‐3↓	Protective effect Promotes cellular immunity	Huang et al. [8]
Ac2‐26	In vivo and in vitro	FPR2/ALX↑ TNF‐α, IL‐6, IL‐1β, MPO↓	Protective effect Inhibits inflammation	Gavins et al. [42]
	In vivo and in vitro	IL‐10↑ IL‐6, TNF‐α ↓	Protective effect Inhibits inflammation	da et al. [44]
	In vivo and in vitro	LXA4↑ p‐PI3K, p‐AKT, NF‐κB, TNF‐α, cleaved caspase‐8/3↓	Protective effect Inhibits inflammation and apoptosis	Zhang et al. [40]
	In vitro	FPR2, Bcl‐2↑ p‐PI3K, p‐AKT, NF‐κB, TNF‐α, IL‐1β, IL‐6, cleaved caspase‐8/3, FADD, Bax↓	Protective effect Inhibits inflammation and apoptosis	Zheng et al. [3]
	In vivo	FPR2, eNOS, IL‐10↑ iNOS, NF‐κB, TNF‐α, IL‐1β, IL‐6, MDA, MPO↓	Protective effect Inhibits inflammation	Chen et al. [43]
ANXA1sp	In vitro	SIRT3, SOD, GSH, GPX4, FTH1↑ TNF‐α, IL‐1β, IL‐6, ROS, MDA, iron, p53, ac‐p53 ↓	Protective effect Inhibits ferroptosis, inflammation, and oxidative stress	Qin et al. [23]
	In vivo and in vitro	DHE, SIRT3, PCG‐1α, TFAM, SOD, ATP, p62↑ TNF‐α, IL‐6, IL‐1β, CK‐MB, cTnI, LDH, ROS, MDA, LC3Ⅱ/LC3Ⅰ, Autophagosomes, autolysosomes↓	Protective effect Promote mitochondrial biosynthesis and inhibit autophagy and oxidative stress	Qin et al. [24]
	In vivo	MMP, ATP, PPAR‐γ, Bcl‐2↑ IL‐6, TNF‐α, ROS, NF‐κB, Bax, Caspase‐3 ↓	Protective effect Inhibit inflammation and apoptosis	Cui et al. [41]
CR‐Ac_2‐50_	In vivo	Neutrophils and monocytes, TNF‐α, IL‐6↓	Protective effect Inhibit inflammation	Gobbetti et al. [38]
Inhibition of SPI1	In vivo and in vitro	ANXA1↑ LC3Ⅱ/LC3Ⅰ↓	Protective effect Inhibit autophagy	Xie et al. [45]
IL‐6	In vivo and in vitro	ANXA1↑ TNF‐α, IL‐1β ↓	Protective effect Inhibit inflammation	de et al. [46]
H2S	In vivo and in vitro	ANXA1, PMN↑ COX2, iNOS, CSE, CD11b↓	Protective effect Inhibit inflammation	Brancaleone et al. [47]

Collectively, ANXA1 and its derived peptide can be protective against sepsis and sepsis‐induced organ damage by improving mitochondrial biosynthesis [24] and inhibiting oxidative stress [23, 24, 39], apoptosis [3, 40, 41], autophagy [24, 39], and ferroptosis [23]. These findings further confirm the potential of ANXA1 as a therapeutic target for sepsis and related diseases. However, most of these studies have focused on a single signaling molecule like SIRT3 [23, 24] and FPR2 [3, 42, 43], and there is a need for future integration of a multi‐omics approach to reveal their synergistic regulatory networks. Meanwhile, most experiments for this disease are based on animal and cellular models, and the clinical translational potential needs to be further confirmed by human sample studies.

## 3 ANXA1 and Hypertension

In September 2023, the World Health Organization (WHO) released the “Global Report on Hypertension,” which defined hypertension as patients with systolic blood pressure ≥ 140 mmHg or diastolic blood pressure ≥ 90 mmHg or who were taking antihypertensive drugs. In addition, reports revealed that the incidence of cardiovascular disease, stroke, and premature death is likely increased by hypertension [48]. Hypertension and its complications affect more than 1 billion people worldwide and remain a major public health problem, highlighting that patients can benefit from strict blood pressure control. Researchers have identified that oxidative stress, immune response, mitochondrial dysfunction [49], and chronic inflammation are the primary pathological mechanisms leading to endothelial damage and vascular stiffness [50], which also affect other tissues and organs.

Recently, a growing number of studies have shown that ANXA1 is closely related to the progression of hypertension [51, 52]. Cardiac and vascular function were measured in different groups of mice (based on age and whether ANXA1 was knocked out), revealing that endogenous ANXA1 plays a key role in lowering blood pressure, improving cardiovascular function, inhibiting vascular remodeling, and delaying cardiac aging [52]. In addition, Potus et al. [49] identified differential genes in rats with pulmonary arterial hypertension and right ventricular failure in 2018, and identified a related dysregulated gene ANXA1, which was presumed to be a potential target for the treatment of pulmonary hypertension with right ventricular failure (RVF–PAH). The ANXA1 gene is upregulated 1.8 times in right ventricular, and transcriptomic analysis reports that ANXA1 is associated with inflammation, fibrosis, mitochondrial metabolism, and angiogenesis in RVF–PAH. Additionally, Mattias et al. analyzed clinical data and found significantly increased expression of ANXA1 in patients with pulmonary arterial hypertension, which was proportional to the severity of the disease, suggesting that ANXA1 may affect the disease process of pulmonary arterial hypertension [53]. Nonetheless, the specific mechanism remains unclear. Zhong et al. reported that reducing the expression level of ANXA1 protein and messenger RNA in rats with the hypothalamus of spontaneously hypertensive rats with hyperactivity of liver‐YANG syndrome can effectively control hypertension and improve hyperactivity [51], indicating that ANXA1 may act as a potential therapeutic target for hypertension.

Interestingly, however, in the study of ANXA1, one study contradicted the above findings. In 2018, Feng et al. found inhibition of ANXA1 and downregulation of the Janus Kinase 2 (JAK2)/STAT3 pathway resulted in a reduction in inflammatory response and apoptosis in a rat model of pre‐eclampsia (PE) characterized by gestational hypertension [54]. This data suggests that ANXA1 may be a damaging molecule in PE. This discrepancy may arise from the unique immune microenvironment of pregnancy or the hormonal milieu specific to gestational hypertension. However, due to insufficient related research, we are currently unable to draw definitive conclusions (Table 3).

**Table 3:** Possible mechanisms of ANXA1 and its derived peptides involved in hypertension.

Administration	In vivo/in vitro model	Findings	Results	Reference
Pinggan Qianyang recipe	In vivo	HSP27↑	Protective effect Inhibit inflammation and apoptosis	Zhong et al. [51]
		ANXA1, TpxⅡ↓		
Inhibition of ANXA1	In vivo and in vitro	Bcl‐2↑	Protective effect Inhibit inflammation and apoptosis	Feng et al. [54]
		p‐JAK2, p‐STAT3, Bax, cleaved caspase‐3, TNF‐α, IL‐1β, IL‐6, IL‐8↓		

Abbreviation: STAT3, signal transducer and activator of transcription 3.

At present, studies on the pathological mechanism of ANXA1 and its derived peptides in hypertension are scarce and have only explored pathological changes and molecular phenotypes, while pathways have been overlooked. The differences in the research progress of pre‐eclampsia and other types of hypertensions suggest that ANXA1 and its derived peptides affect hypertension. However, whether ANXA1 exerts protective or harmful effects and the specific mechanism involved requires further research. Future experiments need to target the functional heterogeneity of ANXA1 in different disease types through conditional knockout technology to explain its paradoxical expression in specific models such as gestational hypertension.

## 4 ANXA1 and Coronary Heart Disease (CHD)

Coronary atherosclerotic heart disease refers to heart disease caused by stenosis or occlusion of the lumen of the coronary artery due to atherosclerosis, resulting in myocardial ischemia, hypoxia, or necrosis, referred to as CHD. This condition is also called ischemic heart disease. In terms of clinical phenotypes, CHD can be further divided into silent myocardial ischemia, stable angina, acute coronary syndrome (unstable angina and myocardial infarction), and sudden cardiac death [55]. As one of the major cardiovascular diseases, CHD is also the leading cause of death in the world [56]. Hence, identifying preventive methods and exploring effective clinical treatment methods are urgent problems to be solved.

Past findings illustrate that ANXA1 can inhibit the onset and progression of CHD and influence disease prognosis. In 2021, researchers reviewed the mechanism of adenosine 5′‐triphosphate (ATP)‐binding cassette subfamily A member 1 (ABCA1) protein in the exocytosis axis and revealed its potential as a novel therapeutic target for atherosclerosis, a major risk factor for CHD. This potential was linked to ANXA1, which inhibits atherosclerosis by promoting the release of anti‐inflammatory cytokines through exocytosis to reduce inflammatory responses in patients [57]. Moreover, another study indicated that ANXA1 in peripheral blood monocytes of patients improved the body's anti‐inflammatory ability and maintained plaque stability by upregulating glucocorticoid sensitivity [58, 59].

In addition to being able to prevent the occurrence of CHD, ANXA1 also plays a role in inhibiting its progression and improving disease prognosis. In a 2019 study, the investigators reported significantly increased ANXA1 levels in the blood of patients with myocardial infarction showing disease progression [60]. Coincidentally, Liu et al. [61] studied the transcriptome of monocytes in CHD patients and identified differentially expressed genes, revealing that the adipokines ANXA1 and SEMA3B were associated with changes in the physiological function of coronary epicardial adipose tissue, thereby participating in the pathogenesis of CHD. These findings suggest that ANXA1 may exist as a potential therapeutic target in the disease progression of myocardial infarction.

Furthermore, Chen et al. [28] found that the ANXA1‐derived peptide RTP‐026 inhibited neutrophil and monocyte activation, as evidenced by CD62L and CD54 expression in patients with myocardial infarction. These changes were associated with reduced neutrophil and monocyte recruitment to the injured cardiac tissues and reduced infarct size of cardiac tissue. Similar inflammatory protective effects were observed in other ANXA1‐derived peptides (Ac2‐26) [62], resulting in improved MI prognosis and reduction of reperfusion injury. Moreover, researchers reported that the presence of ANXA1 can enhance hematopoietic stem/progenitor cell (HSPC) activation, limit excessive monocyte production [63], and result in macrophage polarization [64] to reduce cardiac necrosis, inflammation, hypertrophy, and fibrosis caused by excessive inflammation after myocardial infarction, thereby restoring left ventricular structure and function (Table 4).

In conclusion, ANXA1 plays a crucial role in preventing CHD, inhibiting disease progression, and improving disease prognosis. ANXA1 can also be used to monitor the progression of CHD and evaluate the severity of CHD. At the same time, in the aforementioned study, ANXA1 not only plays a role in inhibiting inflammation, but its impact on adipose tissue suggests that ANXA1 may regulate lipid metabolism, thereby affecting CHD, providing us with a novel research perspective.

## 5 ANXA1 and Myocardial Ischemia–Reperfusion Injury (MIRI)

Ischemia–reperfusion injury (I/RI) refers to a pathological state in which the blood supply of the tissue or organ is restored after a period of ischemia, but the physiological function of the tissue or organ is not restored in time, which results in dysfunction and structural damage. The mechanism of MIRI can be divided into two phases: the ischemic period and the reperfusion period. During ischemia, hypoxia impairs energy metabolism in various tissues, which decreases the intracellular ATP levels and leads to a series of cellular damage, such as disrupted intracellular calcium homeostasis and increased apoptosis [65]. During the reperfusion period, blood flow is re‐established in the damaged tissue area, and blood oxygen supply is increased. However, this process is accompanied by increased production of oxygen‐free radicals, leading to further damage to tissue cells, called reperfusion injury. In addition, reperfusion may activate an inflammatory response, thereby promoting apoptosis and necrosis, damaging cardiomyocytes, and exacerbating tissue or organ I/RI [66].

In recent years, ANXA1 has been recognized and studied as a powerful anti‐inflammatory molecule in the human body and has been found to play a protective role in the process of MIRI [67]. Testai et al. [68] conducted in vitro experiments and reported increased molecular levels of ANXA1 during Erucin (ERU) protective treatment in a MIRI model, accompanied by decreased inflammation, leading to a reduction in the ischemic area and a downregulation of the cTnI index. These findings suggest that ERU may play a protective role in MIRI by upregulating ANXA1. Moreover, ANXA1 can regulate the immune response in the body to decrease acute and chronic inflammation [69]. In addition, a 2021 study revealed that acetylated ANXA1 could promote phosphorylation of caspase‐9 by enhancing the expression of upstream kinases of caspase‐9 after oxygen‐glucose deprivation/R stimulation, thereby reducing the activation of caspase‐3 and inhibiting apoptosis [70]. In addition, ANXA1 can also up‐regulate the molecular level of IL‐1 by increasing the expression of p65, which can further induce the apoptosis of retinal ganglion cells following I/RI [71].

Moreover, some studies revealed that ANXA1‐derived peptides Ac2‐26 can effectively enhanced the survival of cardiomyocytes [72] and maintained their functional stability in the early stage of MIRI by inhibiting the associated inflammatory response, myocardial fibrosis, and apoptosis [62]. Similarly, another ANXA1‐derived peptide, CR‐Ac_2‐50_, has been confirmed to reduce neutrophil‐endothelial interactions, alleviate inflammation, stimulate neutrophil apoptosis and macrophage exocytosis, thereby reducing the area of infarction and lowering the 24‐h mortality rate, providing protective effects on tissues [73]. CR‐Ac_2‐48_, A new type of peptide designed based on the template of CR‐Ac_2‐50_, has been shown to have strong pro‐resolving and tissue‐protective abilities while maintaining high affinity and specificity for FPR2/ALX. In contrast, Ac2‐26 and Ac2‐12 are combined with FPR1 and FPR2 at the same time. CR‐Ac_2‐48_ potent anti‐inflammatory and pro‐resolving effects are observed in phagocytes, which are mediated by activating the p38/AMP‐activated protein kinase (MAPK) pathway and promoting ALX receptor homodimerization [11] (Table 4).

**Table 4:** Possible mechanisms of ANXA1 and its derived peptides involved in CHD/MIRI.

Administration	In vivo/in vitro model	Findings	Results	Reference
Inhibition of ANXA1	In vivo and in vitro	HSPC, NLRP3, TNF‐α, IL‐1β, ROS, CTGF, MMP‐9, cTnI↑ MEP, CMPs, IL‐10↓	Damaging effect Promotes inflammation and vascular remodeling	Qin et al. [63]
RTP‐026	In vivo and in vitro	p‐ERK, p‐AMPK, IL‐1↑ TNF‐α, IL‐1β, IL‐6, KC, VEGF, MIP‐1α, PGE2, PGF2α ↓	Protective effect Inhibit inflammation	Chen et al. [28]

In summary, ANXA1 plays a protective effect in MIRI and is involved in the two major pathological mechanisms leading to MIRI, namely inflammation and apoptosis. At the same time, a 2003 research pointed out that after binding Ca^2+^ the N‐terminal domain of ANXA1 is exposed and undergoes conformational changes [74], after which Dalli et al. found this new structure susceptible to cleavage by proteolytic enzymes [73], including human proteinase (PR) 3 [75] and neutrophil elastase (HNE) [76], leading to inactivation of ANXA1. Therefore, from a therapeutic perspective exploring a cleavage‐resistant AnxA1 or related peptide is necessary. Therefore, from a therapeutic perspective exploring a cleavage‐resistant AnxA1 or related peptide is necessary.

## 6 ANXA1 and Heart Failure (HF)

HF represents the end stage of the development of various cardiovascular diseases. Currently, approximately 40 million people worldwide suffer from HF [77]. In the 2022 American College of Cardiology/American Heart Association (ACC/AHA) guidelines for the management of HF, the condition is defined as a complex clinical syndrome caused by impaired ventricular filling or ejection capacity due to impaired cardiac structure or function, with corresponding symptoms and signs [78]. At present, three main pathological mechanisms have been described in HF: Frank–Starling mechanism, neurohumoral mechanism, and ventricular remodeling [79, 80, 81].

In recent years, ANXA1 has been found to be involved in the regulation of HF. Yu et al. [82] monitored gene expression during the development of HF in a rat model of post‐myocardial infarction, revealing that genes regulating ANXA1 were activated on the first day after myocardial infarction, which may be involved in the subsequent occurrence and development of HF in rats after hyperemia. In patients with acute HF complicated with renal impairment, Adel et al. [83] reported that the elevated level of ANXA1 is often accompanied by more severe hyperemia, increased creatinine levels, and increased mortality, which may be used as a marker of acute HF congestion. Moreover, ANXA1 may inhibit the development of MI. Studies have shown that ANXA1 can promote the polarization of cardiac macrophages to release a large amount of vascular endothelial growth factor (VEGF)‐A [84], thereby inhibiting the inflammatory response and fibrosis of myocardial tissue in mice [85], inducing neovascularization and cardiac repair, and reducing cardiac tissue damage.

In summary, the research on ANXA1 in HF explored its role as a diagnostic marker in clinical practice. Some studies have clarified the protective effect of ANXA1 on HF model mice but only investigated the histopathological and cell phenotype changes, whereas the underlying pathogenesis remains poorly understood.

## 7 ANXA1 and Cerebrovascular Disease (CD)

In the past decade, research on neurological diseases has explored the impact of CD. A growing number of studies have suggested that heart disease is an important factor in the development of CD. Considering the protective effect of ANXA1 in circulatory system diseases, the main studies on ANXA1 in the occurrence and development of CD are summarized below. Ramiro et al. [86] conducted a proteomic and transcriptomic analysis of patients with ischemic stroke and found that ANXA1 was differentially detected in the blood of patients with ischemic stroke in the first week of onset. These findings revealed that ANXA1 may be used as a biomarker or therapeutic target for the diagnosis of patients with ischemic stroke, which requires further exploration.

Therefore, since 2015, researchers have been conducting extensive experiments on the possible effects of ANXA1 and its derivative peptides in CD. Liu et al. conducted in vivo experiments and found that chloral hydrate pretreatment could up‐regulate ANXA1 levels and reduce inflammation levels in mice to prevent ischemic stroke [87]. In addition, Zhou et al. found that the administration of a Tat‐NTS peptide, which prevents ANXA1 from entering the nucleus [88], induced ANXA1 SUMOylation in mouse microglia. On the one hand, SUMO‐modified ANXA1 can inhibit the activation of nuclear factor‐κB through selective autophagy to promote inhibitory Kappa B Kinase α (IKKα) degradation and promote the transformation of microglia to an anti‐inflammatory phenotype, thereby reducing the inflammatory response in cerebral ischemia model mice. This effectively reduces cerebral infarction volume, improves neurological function, and promotes behavioral recovery of ischemic stroke mice [89, 90]. On the other hand, SUMO‐modified ANXA1 can also protect primary neurons from apoptosis in the brain tissue of ischemic stroke mice by downgrading the p53/caspase‐3 pathway. Meanwhile, one study found that dessuccinylation of ANXA1 inhibited the protective effect of SUMOylated ANXA1, which instead led to neuronal cell damage after ischemic stroke [91]. In 2019, Senchenkova et al. conducted in vitro and in vivo experiments [4] and found that ANXA1 gene knockout mice had significantly increased platelet adhesion and aggregate formation in vivo, increasing the risk of stroke; ANXA1 administration could directly or indirectly regulate platelet function in mice with cerebral I/RI. Reducing thromboxane B and regulating phosphatidylserine expression to inhibit platelet activation, aggregate formation, and cerebral thrombosis can be used as a therapeutic drug or a preventive drug to protect against cerebral I/RI. Xu et al. [6] monitored plasma ANXA1 as a potential biomarker for the prognosis of endovascular thrombectomy (EVT) in patients with acute ischemic stroke (AIS). Further experiments showed that Ac2‐26 could activate the FPR2/ALX‐dependent AMP‐activated protein kinase ‐ mammalian target of rapamycin pathway similarly to ANXA1 [92], and this effect promotes the polarization of microglia/macrophages while inhibiting the inflammatory response in cerebral I/RI. Furthermore, the ANXA1 mimetic peptide Ac2‐26 can be used as an auxiliary strategy for clinical prevention and treatment of cerebral I/R injury in AIS patients after EVT treatment. ANXA1 also plays a certain role in patients with spontaneous intracerebral hemorrhage (ICH). Researchers found that ANXA1 combined with the FPR2 receptor can down‐regulate the p38 signaling molecule, effectively reduce brain edema after ICH, improve microglia activation, and restore short‐term neurological function of patients. These results hint that ANXA1 may be a potential therapeutic strategy for ICH patients.

However, the presence of ANXA1 is not necessarily beneficial. In 2022, a study [93] rescued synaptic damage and cognitive impairment caused by transient ischemic attack (TIA) with a multiple mild stimulation (MMS) technique, which was closely related to the presence of ANXA1. Studies have found that ANXA1 in microglia promotes the binding of CX3CR1 in microglia to CX3CL1 in neurons during TIA, reduces the density of dendritic spines, and leads to synaptic damage and cognitive impairment caused by TIA. The MMS technology can significantly inhibit this process (Table 5).

**Table 5:** Possible mechanisms of ANXA1 and its derived peptides involved in CD.

Administration	In vivo/in vitro model	Findings	Results	Reference
ANXA1	In vivo	αIIbβ3, p‐AKT, Ca^2+^, Rap1, PNA, TXA2↓	Protective effect Inhibit inflammation	Senchenkova et al. [4]
	In vivo	p38, COX‐2, TNF‐α, IL‐1β, cPLA‐2↓	Protective effect Inhibit inflammation	Ding et al. [94]
	In vivo	FPR2, p‐ERK, DUSP1, CD36 ↑ TNF‐α ↓	Protective effect Inhibit inflammation	Flores et al. [95]
SUMOylated ANXA1	In vivo and in vitro	Autophagy vacuoles, Arg‐1, IL‐4, IL‐10, TGF‐β, CD206↑ p65, TNF‐α, IL‐1β, IL‐6, iNOS, CD16/32, IKKα ↓	Protective effect Inhibit inflammation	Li et al. [89]
Ac2‐26	In vivo and in vitro	p‐AMPAα, IL‐10, CD206, Arg‐1, YM1↑ p‐mTOR, IL‐1β, Iba‐1, CD16/32, iNOS, cleaved caspase‐3↓	Protective effect Inhibit inflammation and apoptosis	Xu et al. [6]
Tat‐NTS peptide	In vivo and in vitro	ANXA1, p53, caspase‐3/9, cleaved PARP, Bid↓	Protective effect Inhibit apoptosis	Li et al. [88]
	In vivo and in vitro	SUMOylated ANXA1↑ IKKα, p65, TNF‐α, IL‐1β ↓	Protective effect Inhibit inflammation and apoptosis	Zhou et al. [90]
SENP6	In vivo and in vitro	TRPM7, PKC, p53, Bid, cleaved caspase‐3/9, cleaved PARP, LDH↑ SUMOylated ANXA1↓	Damaging effect Promote apoptosis	Xia et al. [96]
SIRT5	In vivo and in vitro	caspase‐3/9, cleaved PARP, LDH, TNF‐α, IL‐1β, IL‐6, iNOS, Cxcl1, Ccl2, Iba‐1↑ SUMOylated ANXA1↓	Damaging effect Promote inflammation and apoptosis	Xia et al. [91]
Electroacupunc‐ture (EA)	In vivo	Arg‐1, BDNF↑ TNF‐α, IL‐1β, iNOS↓	Protective effect Inhibit inflammation	Zou et al. [97]
Chloral hydrate	In vivo	ANXA1, IL‐4, IL‐10, TGF‐β ↑ TNF‐α, IL‐1β, IL‐6↓	Protective effect Inhibit inflammation	Liu et al. [87]
Multiple mild stimulations (MMS)	In vivo	ANXA1, CX3CR1, CX3CL1, PSD95↓	Protective effect Inhibit the over‐ pruning of synaptic spines	Zheng et al. [93]

Abbreviation: NTS, nuclear translocation signal.

In conclusion, ANXA1 plays a powerful and extensive role in the clinical diagnosis and treatment of CD. ANXA1 behaves as a protective molecule in most circulatory diseases but promotes synaptic damage in TIA [93], suggesting an environment‐dependent role. This bi‐directionality may be related to the tissue‐specific expression of FPR receptor subtypes or reflect their different roles in acute injury versus chronic repair.

## 8 ANXA1 and Other Circulatory Diseases

### 8.1 Peripheral Vascular Disease (PVD)

PVD is an increasingly prominent health problem. Its high prevalence is attributed to obesity, diabetes, and population aging, with more than 200 million persons affected worldwide [98]. According to the involved vessels, the condition is classified as venous, arterial, or mixed.

Since 2022, researchers have found that ANXA1 can partially affect related PVD. In 2023, Tian et al. [99] used single‐cell RNA sequencing technology for cell‐to‐cell communication analysis and found that ANXA1 was differentially expressed in ascending thoracic aortic aneurysm (ATAA) patients and ordinary people as a target molecule associated with immunogenic cell death in endothelial cells. Hence, ANXA1 may be involved in regulating the process of disease development. In addition, ANXA1 primarily binds to the FPR1 receptor and affects endothelial cells to act on monocytes and macrophages.

Acute aortic dissection (AAD) is the most common fatal disease in acute aortic syndrome. According to the latest report published by the International Aortic Dissection Registry (IRAD), the overall mortality rate of AAD is 35.8% [100]. Despite the high mortality associated with AAD, there are no risk factors that predict AAD [101]. Recently, Zhou et al. [102] revealed that the level of ANXA1 was significantly increased in the AAD group compared to the control one. Thus, ANXA1 may be a marker for the diagnosis of AAD and may be involved in the occurrence and development of AAD. After the Ac2‐26 intervention, ANXA1 was found to inhibit the phenotypic switching of smooth muscle cells in vivo by activating the JunB/MYL9 pathway. Furthermore, the reduction of matrix metalloproteinase (MMP) production also plays a role in protecting aortic elastin from degradation (Table 6).

**Table 6:** Possible mechanisms of ANXA1 and its derived peptides involved in other circulatory diseases.

Administration	In vivo/In vitro model	Findings	Results	Reference
Inhibition of ANXA1	In vivo	TNF‐α, IL‐6, MYH11, SMA, Calponin, SM22α, MMPs↑ JunB, MYL9↓	Protective effect Inhibit inflammation and vascular remodeling	Zhou et al. [102]

Despite these findings, several gaps remain. The specific mechanisms by which ANXA1 regulates endothelial and immune cell interactions in different types of PVD need further elucidation. Additionally, the long‐term effects of ANXA1‐derived peptide, such as Ac2‐26, on vascular function and remodeling require more comprehensive studies. Future research should focus on validating ANXA1 as a therapeutic target through large‐scale clinical trials and exploring its role in other cardiovascular diseases.

### 8.2 Coronary Artery Aneurysm (CAA)

Coronary artery ectasia (CAE) or CAA is a rare cardiovascular disease. Its incidence ranges from 1.2% to 4.9% [103]. Coronary artery dilatation is generally a dilatative vascular remodeling (excessive dilatative remodeling) in response to the growth of atherosclerotic plaques. Hypertension is a high‐risk factor, and atherosclerosis, Kawasaki disease, and congenital heart disease are the most common causes. However, its pathological mechanism remains unclear. Weng et al. [104] analyzed clinical data in patients suffering from Kawasaki disease with CAA and found reduced serum ANXA1 levels; serum ANXA1 levels were positively correlated with erythrocyte sedimentation rate (ESR), IL‐6, and D‐dimer (DD) levels, which may lead to the hypercoagulable state of Kawasaki disease.

These findings indicate that ANXA1 may be involved in the occurrence and development of Kawasaki disease with CAA; however, the pathological mechanisms of CAE/CAA remain unclear. Future research should focus on elucidating the role of ANXA1 in disease progression and its potential as a biomarker or therapeutic target. Additionally, exploring the underlying mechanisms linking ANXA1 to inflammation and coagulation in these diseases is essential.

## 9 Discussion

As an endogenous anti‐inflammatory protein, ANXA1 plays a multifaceted role as a regulator in a variety of circulatory diseases, exhibiting different biological effects depending on the disease. Numerous studies have highlighted its role in the regulation of inflammation, apoptosis, immune response and vascular remodeling, and have positioned it as a potential therapeutic target and biomarker.

In sepsis and septic shock, ANXA1 and its derivative peptides show great potential in reducing inflammation and improving prognosis. Studies have identified several mechanisms such as activation of anti‐inflammatory pathways and inhibition of pro‐inflammatory cytokines like TNF‐α and IL‐6 by interacting with receptors such as FPR2/ALX [3, 42, 43]. However, most studies have focused on individual signaling molecules, and a comprehensive understanding of the coregulatory network involving ANXA1 remains elusive. Future studies should adopt a multi‐omics approach to elucidate these interactions and validate the findings in human clinical trials.

In hypertension, ANXA1 has been implicated in both protective and detrimental roles. While some studies suggest that ANXA1 helps lower blood pressure and inhibit vascular remodeling [52], others indicate that its inhibition may reduce inflammation and apoptosis [54] in specific conditions like pre‐eclampsia. This discrepancy highlights the need for further investigation into the functional heterogeneity of ANXA1 in different hypertension models, particularly through conditional knockout studies and comparisons of gestational versus nonpregnant hypertension.

For CHD and MIRI, ANXA1 has consistently demonstrated protective effects. It inhibits inflammation [28, 69], reduces apoptosis [70], and promotes macrophage polarization [64] and neovascularization [63]. Derived peptides like Ac2‐26 [62, 72] and CR‐Ac2‐50 [73] have shown promise in reducing infarct size and improving cardiac function. However, the long‐term effects of these interventions and their impact on lipid metabolism require further exploration.

In CD, ANXA1 has shown protective effects in ischemic stroke and intracerebral hemorrhage by modulating inflammation [89, 90] and platelet function [4]. However, its role in TIA is more complex, with findings suggesting that ANXA1 may contribute to synaptic damage [93] and cognitive impairment. This highlights the context‐dependent nature of ANXA1's effects and underscores the need for a nuanced understanding of its mechanisms in different disease settings.

In PVD and CAA, ANXA1 has been identified as a potential biomarker [101] and therapeutic target [104]. However, the specific mechanisms underlying its involvement in these diseases remain unclear. Further research is needed to elucidate how ANXA1 regulates endothelial and immune cell interactions and to explore its long‐term effects on vascular remodeling.

In summary, ANXA1 is a promising candidate for therapeutic intervention in various circulatory diseases. However, its effects are highly context‐dependent, and further research is needed to fully understand its mechanisms and optimize its clinical application. Future studies should focus on multi‐omics approaches to unravel the complex regulatory networks involving ANXA1, validate its therapeutic potential through large‐scale clinical trials, and explore its role in diverse disease settings.

## 10 Summary and Perspective

The study of ANXA1 in circulatory diseases has revealed its multifaceted role as a potential therapeutic target and biomarker. As research continues to uncover the mechanisms by which ANXA1 modulates inflammation, apoptosis, and immune responses, its clinical application in various cardiovascular and CD becomes increasingly promising. However, the context‐dependent nature of ANXA1's effects highlights the complexity of its role in disease progression and resolution. Future research should focus on elucidating the intricate regulatory networks involving ANXA1 through multi‐omics approaches, which could provide a comprehensive understanding of its interactions with other signaling pathways. Additionally, large‐scale clinical trials are essential to validate the therapeutic potential of ANXA1 and its derived peptides, ensuring their efficacy and safety in diverse patient populations. Given the growing prevalence of circulatory diseases, further exploration of ANXA1's role in disease prevention, diagnosis, and treatment is of utmost importance.

## Author Contributions

**Qiuyu Dai:** conceptualization, writing – original draft. **Jie Zheng:** writing – original draft. **Meng Fu:** writing – original draft. **Song Qin:** conceptualization, funding acquisition, writing – review and editing. **Xiaoyun Fu:** funding acquisition, supervision, writing – review and editing.

## Conflicts of Interest

The authors declare no conflicts of interest.

## Data Availability

The datasets used and/or analyzed during the current study are available from the corresponding author on reasonable request.
